# Using traditional machine learning and deep learning methods for on- and off-target prediction in CRISPR/Cas9: a review

**DOI:** 10.1093/bib/bbad131

**Published:** 2023-04-20

**Authors:** Zeinab Sherkatghanad, Moloud Abdar, Jeremy Charlier, Vladimir Makarenkov

**Affiliations:** Departement d’Informatique, Universite du Quebec a Montreal, H2X 3Y7, Montreal, QC, Canada; Institute for Intelligent Systems Research and Innovation (IISRI), Deakin University, 3216, Geelong, VIC, Australia; Departement d’Informatique, Universite du Quebec a Montreal, H2X 3Y7, Montreal, QC, Canada; Departement d’Informatique, Universite du Quebec a Montreal, H2X 3Y7, Montreal, QC, Canada

**Keywords:** CRISPR-Cas9, Genome Editing, Machine Learning, Deep Learning, On-Targets, Off-Targets

## Abstract

CRISPR/Cas9 (Clustered Regularly Interspaced Short Palindromic Repeats and CRISPR-associated protein 9) is a popular and effective two-component technology used for targeted genetic manipulation. It is currently the most versatile and accurate method of gene and genome editing, which benefits from a large variety of practical applications. For example, in biomedicine, it has been used in research related to cancer, virus infections, pathogen detection, and genetic diseases. Current CRISPR/Cas9 research is based on data-driven models for on- and off-target prediction as a cleavage may occur at non-target sequence locations. Nowadays, conventional machine learning and deep learning methods are applied on a regular basis to accurately predict on-target knockout efficacy and off-target profile of given single-guide RNAs (sgRNAs). In this paper, we present an overview and a comparative analysis of traditional machine learning and deep learning models used in CRISPR/Cas9. We highlight the key research challenges and directions associated with target activity prediction. We discuss recent advances in the sgRNA–DNA sequence encoding used in state-of-the-art on- and off-target prediction models. Furthermore, we present the most popular deep learning neural network architectures used in CRISPR/Cas9 prediction models. Finally, we summarize the existing challenges and discuss possible future investigations in the field of on- and off-target prediction. Our paper provides valuable support for academic and industrial researchers interested in the application of machine learning methods in the field of CRISPR/Cas9 genome editing.

## INTRODUCTION

Advances in the area of genome editing (also called gene editing) in the 2010s revolutionized molecular biology, genetics, and biomedicine. Genome editing techniques allow precise manipulation, deletion, and insertion of sequence fragments within the DNA of living organisms. In recent years, three effective types of genome editing toolsets, called Zinc Finger Nucleases (ZFNs) [1], Transcription Activator-Like Effector Nucleases (TALENs), and Clustered Regularly Interspaced Short Palindromic Repeats (CRISPR), have been developed to study the process of target modifications in gene sequences [1–5].

**Figure 1:**
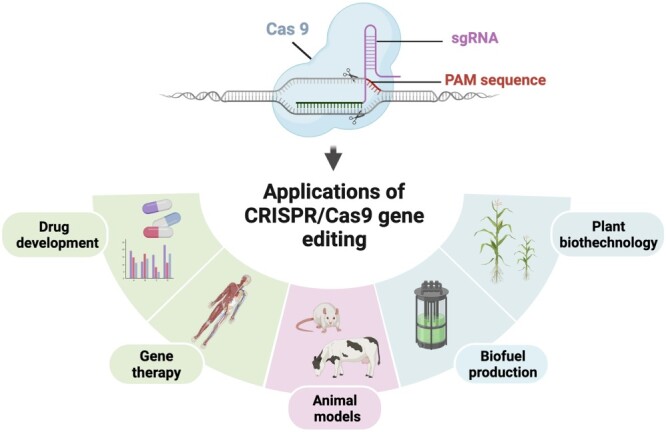
Schematic view of CRISPR/Cas9 gene editing system and its practical applications.

Highly effective CRISPR/Cas9 gene editing system, co-invented in 2012 by Emmanuelle Charpentier and Jennifer Doudna [6], has been used in various fields, ranging from basic research on genetic therapies at the cellular level to applied biomedical research [6–11]. CRISPR/Cas9 demonstrated important clinical potential for treating human diseases such as cancer and genetic disorders [12–14], for plant genetic engineering [15–17], as well as for animal disease treatment [18, 19]. Figure 1 presents a schematic view of the CRISPR/Cas9 gene editing system and its practical applications.

The CRISPR/Cas9 genetic engineering system is an adapted version of the bacterial CRISPR-Cas9 antiviral defense system. CRISPR is a family of DNA sequences present in prokaryotic genomes that stems from DNA fragments of bacteriophages, which had infected prokaryotic genomes in the past. These DNAs are used as antiviral defense elements to recognize and eliminate DNA from similar bacteriophages during eventual infections [20]. Cas9 is a type of nuclease enzyme that uses CRISPR sequences as a guide to identify and cleave specific DNA fragments that are complementary to a given CRISPR sequence.

In the CRISPR/Cas9 editing system, the Cas9 nuclease combined with a guide RNA (gRNA) is delivered into a cell, allowing the cell’s genome to be cut in a specific location, some targeted genes to be removed from it, and some other added to it, *in vivo* [21]. Guide RNA, or artificially programmed single-guide RNA (sgRNA) used in type II CRISPR/Cas9 systems, is responsible for identifying the target DNA sequence in the cell’s genome and ensuring that the cutting takes place at the desired sequence location. The Protospacer-Adjacent Motif (PAM), located at the end of the DNA target site, is a short three-to-five nucleobase sequence serving as the binding signal of the Cas protein [22].

The CRISPR/Cas9 system is nonetheless prone to unintended off-targets: a cleavage may occur at non-target locations [23–25]. Thus, the safety aspect of the use of CRISPR/Cas9 on humans remains an open issue. The main challenge in the effective application of the CRISPR/Cas9 system is to maximize on-target activity (i.e. guide efficiency) and minimize the number of potential off-targets (i.e. guide specificity).

Over the last few years, data-driven machine learning methods emerged as a new modeling approach which outperforms the common scoring prediction methods, such as MIT CRISPR Design Tool2 [26], CCTop algorithm [27], CRISPR Design [28], E-CRISP [29], and CHOPCHOP [30]. One of the main drawbacks of the latter methods is the lack of capacity to increase the prediction accuracy when the number of samples increases. In contrast, one of the main advantages of modern data-driven models relying on deep learning is their ability to improve the predictive performance as the number of samples grows. Current state-of-the-art research aiming at designing robust clinical CRISPR/Cas9 applications looks for enhancing data-driven models by: (i) increasing on-target efficiency, (ii) improving off-target specificity, and (iii) simultaneously maximizing on-target activity and minimizing off-target effects.

Among recent reviews and comparative studies discussing the use of computational methods for evaluating gRNA efficiency and predicting gRNA specificity, we need to mention the following works. Wang *et al*. [31] highlighted new advances in CRISPR-Cas systems in terms of RNA targeting, tracking, and editing. The authors compared Cas protein-based technologies with traditional technologies intended for these goals. Liu *et al*. [32] provided an up-to-date overview of computational methods for gRNA design, including web-based platforms, to help researchers select optimal tools for their CRISPR-Cas experiments. However, among 14 methods for evaluating gRNA efficiency and 13 methods for predicting gRNA specificity considered by Liu *et al*., only one employs deep learning. Chen *et al*. [33] first briefly reviewed the main properties of CRISPR systems and their use in genome editing. Then, the authors discussed feasible methods for detecting potential off-targets during CRISPR/Cas9 genetic manipulations. Yan *et al*. [34] compared 17 *in silico* off-target prediction tools in order to evaluate their genome-wide CRISPR performances, and introduced an integrated Genome-Wide Off-target cleavage Search (iGWOS) platform designed for optimal genome-wide off-target predictions. The main goal of the study by Yaish *et al*. [35] was to systematically evaluate data pre-processing and formulation of the CRISPR off-target prediction problem. The authors pointed out that data transformation is a crucial data pre-processing step which should be applied prior to model training. They highlighted the importance of considering as model’s features both inactive off-target sites and the number of mismatches between gRNAs and their off-target sites. Moreover, Yaish *et al*. introduced predictive off-target *in cellula* models based on gradient boosting (i.e. the XGBoost decision tree-based ensemble learning framework was implemented by these authors) and compared them with state-of-the-art off-target prediction methods. The paper of O’Brien *et al*. [36] presents the main machine learning approaches and pitfalls in the context of CRISPR-Cas9 experiments. The authors consider the related computational problems, including algorithm choice, accuracy overestimation, and data interoperability. They concluded that due to the broad availability of machine learning-based tools, *in silico* optimization can successfully replace *in vitro* CRISPR-Cas9 designs. Thus, algorithmic solutions can be used for maximizing gRNA editing efficiency and minimizing gRNA specificity. Finally, a recent review of Konstantakos *et al*. [37] addresses the problem of CRISPR–Cas9 gRNA efficiency prediction and evaluates the role of deep learning in this context. The authors discussed the main computational approaches for on-target activity prediction, focusing on the selection of optimal features and algorithms. In their comparative experiments, Konstantakos *et al*. assessed the performances of 10 deep learning and one conventional machine learning methods on six benchmark data sets, and provided recommendations for their use. The authors pointed out that the existing on-target prediction approaches still have some flaws, including their sensitivity to data heterogeneity, unclear decision making mechanism, and inability to produce general gRNA design rules.

In this review paper, we provide a summary of studies that have examined the effectiveness of artificial intelligence (AI) methods for on- and off-target activity prediction related to CRISPR/Cas9. In contrast to some previous reviews [32, 34, 37–42], our study discusses the use of both traditional machine learning and deep learning methods, focusing on the latest state-of-the-art on- and off-target prediction models. We describe the main advantages and disadvantage of existing prediction models, while highlighting noticeable progress that has been made in sequence encoding.

Our main contributions are as follows:

- To the best of our knowledge, this is the first comprehensive review of both traditional machine learning and deep learning methods, used for both on-target (guide efficiency) and off-target (guide specificity) outcome prediction in CRISPR/Cas9 genome editing;
- A description of the benchmark data sets used for on- and off-target prediction in CRISPR/Cas9 is provided. Most of the discussed data sets were curated and made available for researchers on our GitHub repository;
- The main sequence encoding techniques used in CRISPR/Cas9, applying to both traditional machine learning and deep learning methods, are discussed along with their intrinsic properties;
- The main deep learning models used for on- and off-target prediction in CRISPR/Cas9 are presented and their advantages and limitations are highlighted;
- Research challenges and avenues of future investigation regarding the application of traditional machine learning and deep learning methods in the field of CRISPR/Cas9 genome editing are discussed.

## DATA DESCRIPTION

In this section, we present the most popular CRISPR/Cas9 benchmark data sets used in the literature for on- and off-target prediction. These data sets can be divided into three categories: data sets including off-targets only, on-targets only, and both off- and on-targets.

The first group of benchmark data sets consists of off-targets. GUIDE-seq was one of the first off-target data repositories, based on the results of the GUIDE-seq technique developed by Tsai *et al*. [43]. It can serve as an accurate framework for genome-wide identification of off-target effects. The sgRNAs used in GUIDE-seq target the following sites: VEGFA site 1, VEGFA site 2, VEGFA site 3, FANCF, HEK293 site 2, HEK293 site 3, and HEK293 site 4, in which 28 off-targets with a minimum modification frequency of $0.1$ were identified (among 403 potential off-targets). The CIRCLE-Seq (Circularization for *in vitro* Reporting of CLeavage Effects by sequencing) screening strategy introduced by Tsai *et al*. [44] was used to analyze the related data set that includes gRNA–DNA pairs for 10 gRNA sequences with the corresponding mismatch, insertion, and deletion information; 7371 of these sequence pairs were identified as active off-targets. Cameron *et al*. [45] proposed the SITE-Seq biochemical method that uses Cas9 programmed with sgRNAs to recognize cut sites within genomic DNA. The related data set contains sgRNA–DNA sequence pairs for nine guide sequences; 3767 of these sequence pairs correspond to active off-targets. Abadi *et al*. [46] collected a training data set based on three genome-wide methods for unbiased CRISPR-Cas9 cleavage site profiling, which are as follows: (i) Genome-wide unbiased identification of DSBs enabled by sequencing (GUIDE-Seq) [43, 47], (ii) High-throughput genome-wide translocation sequencing (HTGTS) [48], and (iii) Breaks labeling, enrichment on streptavidin and next-generation sequencing (BLESS) [49, 50]. The resulting data set was assembled from the five following studies: [43, 47–50]. It includes 33 collections of sgRNAs with their respective targets. Altogether, these sgRNAs cleaved 872 genomic targets across human genome. Lazzarotto *et al*. [51] applied their CHANGE-seq automatable tagmentation-based method to analyze the related *in vitro* Cas9 genome-wide nuclease activity data set. CHANGE-seq was carried out to analyze 110 sgRNA targets across 13 therapeutically relevant loci in human primary T cells. A total of 201 934 off-target sites were identified with variable numbers of off-target sites, ranging from 19 to 61 415, for an individual sgRNA.

The second group of benchmark data sets consists of on-targets. Wang *et al*. [52] used a practical library containing $73\,000$ sgRNAs to generate knockout collections and to investigate screens in human cell lines HL-$60$ and KBM$7$. The authors tested both ribosomal and non-ribosomal protein coding genes with all possible sgRNAs gathered in the library. Koike-Yusa *et al*. [53] conducted their investigation on a data set that consisted of $87\,897$ gRNAs targeting $19\,150$ mouse protein-coding genes. They designed genome-wide mutant mouse embryonic stem cell libraries to identify unknown host factors that modulate toxin susceptibility. The Doench V1 data set [54] consists of 1831 guides targeting three human (CD13, CD15, and CD33) and six mouse (Cd5, Cd28, H2-K, Cd45, Thy1, and Cd43) genes, all producing cell-surface markers, which could be assayed by flow cytometry. GenomeCRISPR is a well-formatted data repository, organized by Rauscher *et al*. [55], which was designed for high-throughput CRISPR screening studies. GenomeCRISPR contains over 550 000 sgRNAs on-targets derived from 84 different experiments. Wang *et al*. [56] used the DeepHf (Deep learning for High-Fidelity Cas9) method to perform a genome-scale screen measuring gRNA activity of two highly specific SpCas9 variants (eSpCas9(1.1) and SpCas9-HF1), and a wild-type SpCas9 (WT-SpCas9) in human cells. The obtained data set contains indel rates for over 50 000 gRNAs for each nuclease, covering about 20 000 genes. It is the largest gRNA on-target activity data set reported to date for mammalian cells. Kim *et al*. [57] generated a data set of SpCas9 activities at 12 832 target sequences from a human cell library using the deep learning–based DeepSpCas9 model. The DeepSpCas9 target sequences were chosen from the human genome and synthetic sequences without using any information related to the activity of the associated sgRNAs. The sgDesigner data are a unique plasmid target library expressed in human cells that was used by Hiranniramol *et al*. [58] for experimental quantification of sgRNA CRISPR/Cas9 efficiency. A pool of 12 472 oligonucleotides was used to train a machine learning algorithm for assay design.

The third group of benchmark data sets considered in the literature includes data containing both off- and on-target information. First, we need to mention the RESistance assays (RES) data set, i.e. Doench V2, made available by Doench *et al*. [59]. It consists of 2549 unique guides targeting eight genes (i.e. CCDC101, MED12, TADA2B, TADA1, HPRT, CUL3, NF1, and NF2) from Human A375 cells. The well-known CRISPOR database organized and maintained by Haeussler *et al*. [60] aggregates different public data sets that have been widely used to quantify on-target guide efficiency and detect off-target cleavage sites, including the Wang-Xu *et al*. data set (2076 guides targeting 221 genes in Human HL-60 cells) [52, 61], Koike-Yusa *et al*. data set [53], Doench V1 and V2 data sets [54, 59], Hart *et al*. data set (4239 guides targeting 829 genes in Human Hct116 cells) [62], Z_fish MM data set (1020 guides targeting 128 genes in Zebrafish genome) [63], Z_fish VZ data set (102 guides targeting different genes in Zebrafish genome) [64], Z_fish GZ data set (111 guides targeting different genes in Zebrafish genome) [65], Drosophila data set [66], Chari *et al*. data set (1234 guides targeting Human 293T cells) [67], Ciona data set (72 guides targeting different genes in Ciona genome) [68], and Farboud *et al*. data set (50 guides targeting different genes in Caenorhabditis elegans genome) [69]. Moreover, Haeussler *et al*. [60] developed the CRISPOR web tool (available at: crispor.org) that is intended to design, evaluate and clone guide sequences for the CRISPR/Cas9 system. This web tool incorporates several on- and off-target scoring algorithms. It also displays pre-calculated results for all human exons from the UCSC Genome Browser tracks. On the first page of crispor.org, the user enters three pieces of information: (i) a single genomic sequence, typically an exon under 2300 bp; (ii) a genome (from the list of more than 150 genomes, including plants and emerging model organisms); (iii) a PAM motif. The main output of CRISPOR is a web page that shows the annotated input sequence and the list of possible guides in this sequence. Furthermore, CRISPOR also generates a list of primers related to a selected guide. The related GitHub data repository organized by Haeussler comprises direct links to 22 experimental data sets along with some necessary data conversion scripts written in Python. Munoz *et al*. [70] designed the CRISPR tiling library, which is a large tiling-sgRNA data set containing data for 139 genes with an average of 364 sgRNAs/gene for three cancer cell lines DLD1, RKO, and NCI-H1299. Furthermore, the DeepCRISPR data set generated by Chuai *et al*. [71] includes approximately $0.68$ billion sgRNA sequences derived from 13 human cell lines, including HEK293, MCF-7, K562, HL60, NB4, BE2C, Caco-2, GM06990, Hela, HCT116, LNCap, HepG2, and GM12878. This large data set comprises epigenetic information for different cell types, providing a unified feature space which combines the data from various experiments and cell types. The related DeepCRISPR software includes the models for both sgRNA on-target knockout efficacy and genome-wide off-target cleavage profile prediction.

In Table 1, we present a summary of the main features of the CRISPR/Cas9 benchmark data sets used for on- and off-target prediction, including the original study describing the data set in question, the URL link to the data set and the target type. We curated the benchmark data reported in Table 1 and made them available for researchers on our GitHub repository at the following URL address: https://github.com/dagrate/public_data_crisprCas9. Moreover, several data sets presented here have been one-hot encoded and prepared for use in machine learning and deep learning experiments. The related Python scripts have been also made available to the scientific community.

**Table 1:** A summary of the most popular CRISPR/Cas9 benchmark data sets and databases used for on- and off-target prediction.

Source	Year	Data description	Target	Data link
Wang *et al*. data [52]	2014	A library containing $73\,000$ sgRNAs	On-targets	https://www.ncbi.nlm.nih.gov/pmc/articles/PMC3972032/#SD2
Koike-Yusa *et al*. data [53]	2014	$87,897$ gRNAs targeting $19\,150$ mouse protein-coding genes	On-targets	Deposited at the European Nucleotide Archive under accession number ERP003292.
Doench V1 data [54]	2014	1831 guides targeting three human (CD13, CD15 and CD33) and six mouse genes (Cd5, Cd28, H2-K, Cd45, Thy1 and Cd43)	On-targets	broadinstitute.org/rnai/public/analysis-tools/sgrna-design
GUIDE-seq data [43]	2015	CRISPR RNA-guided nucleases (RGNs) from two human cell lines: U2OS and HEK293; different sites such as VEGFA sites 1, 2 and 3, and HEK293 sites 2, 3 and 4 were studied	Off-targets	https://github.com/tsailabSJ/guideseq
Doench V2 data [59]	2016	2549 unique guides targeting eight genes (CCDC101, MED12, TADA2B, TADA1, HPRT, CUL3, NF1 and NF2) from human A375 cells	Off-targets and on-targets	https://www.nature.com/articles/nbt.3437
CRISPOR program + data repository [60]	2016	Aggregate data for more than 150 genomes, including the following public data sets: Wang-Xu [52, 61], Koike-Yusa [53], Doench V1 and V2 [54, 59], Hart [62], Z_fish MM [63], Z_fish VZ [64], Z_fish GZ [65], osophila [66], Chari [67], Dr Ciona [68], Farboud [69]	Off-targets and on-targets	http://crispor.org + https://github.com/maximilianh/crisporPaper/tree/master/effData#readme
GenomeCRISPR	2016	Aggregate data for more than 550 000 sgRNAs derived from 84 experiments	On-targets	http://genomecrispr.org
database [55]				
CIRCLE-Seq data [44]	2017	Contains mismatch, insertion and deletion information, and includes sgRNA–DNA pairs from 10 guide sequences, 7371 of which are off-targets (430 with bulges)	Off-targets	https://github.com/tsailabSJ/circleseq
SITE-Seq data [45]	2017	gRNA–DNA pairs from nine guide sequences, 3767 of which are active off-targets (no bulges)	Off-targets	https://experiments.springernature.com/articles/10.1038/nmeth.4284
Abadi *et al*. [46]	2017	A data set based on three genome-wide methods for unbiased CRISPR-Cas9 cleavage sites profiling: GUIDE-Seq, HTGTS and BLESS. It includes 33 collections of sgRNAs with their respective targets	Off-targets	https://journals.plos.org/ploscompbiol/article?id=10.1371/journal.pcbi.1005807
DeepCRISPR [71] platform	2018	Includes approximately $0.68$ billion sgRNA sequences derived from 13 human cell lines	Off-targets and on-targets	https://github.com/bm2-lab/DeepCRISPR
DeepHf data [56]	2019	Includes indel rates of over 50 000 gRNAs for each nuclease, covering about 20 000 genes. It is the largest gRNA on-target activity set reported for mammalian cells	On-targets	http://www.DeepHF.com
DeepSpCas9 data [57]	2019	A dataset of SpCas9 activities at 12 832 integrated target sequences for a human cell library	On-targets	http://deepcrispr.info/DeepSpCas9
sgDesigner data [58]	2020	A unique plasmid library expressed in human cells was used to quantify the potency of thousands of CRISPR/Cas9 sgRNAs (a pool of 12 472 oligonucleotides was analyzed)	On-targets	https://academic.oup.com/bioinformatics/article/36/9/2684/5714741?login=false#supplementary-data
CHANGE-seq data [51]	2020	110 sgRNA targets across 13 therapeutically relevant loci in human primary T-cells were studied to identify 201 934 off-target sites across the human genome	Off-targets	https://github.com/tsailabSJ/changeseq

In conclusion, we think that using the latest benchmark data containing large amounts of samples and features is likely to facilitate future work in CRISPR/Cas9, since such data can provide wide and complete coverage of intrinsic properties of both off- and on-targets under study, and thus be effectively exploited by state-of-the-art machine learning and deep learning methods.

## sgRNA–DNA SEQUENCE ENCODING

**Figure 2:**
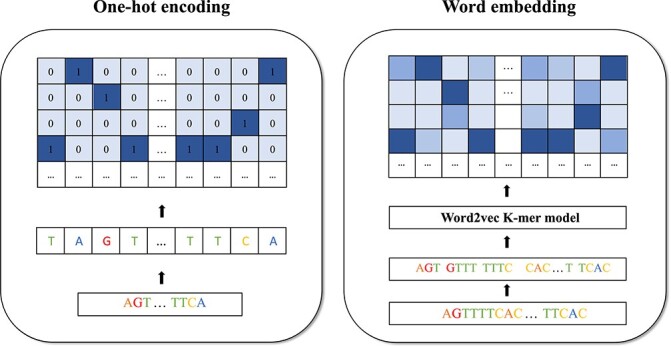
Two sequence encoding models used in CRISPR/Cas9: one-hot encoding and word embedding.

Before building AI models intended for on- and off-target prediction, the sgRNA–DNA sequence data must be pre-processed to be used as input. Data pre-processing, or data encoding, allows converting the sgRNA–DNA sequences of letters into sequences of numbers that AI models can read and interpret to build their predictions. Data pre-processing is an important milestone when trying to boost the predictive performance of AI models. The two most popular encoding techniques used in CRISPR-Cas9 are: (a) One-hot encoding and (b) Word embedding. Figure 2 highlights the differences between the two techniques. In one-hot encoding, each possible channel A, C, G or T is represented by a one-hot vector such as [1,0,0,0], [0,1,0,0], [0,0,1,0], and [0,0,0,1]. In embedding, a particular word, or string, is represented using a unique vector representation. A sgRNA–DNA sequence, which can be subdivided into substrings of length $k$, called $k$-mers, can be thus transformed into a vector representation. The most popular embedding technique is Word2Vec [72]. This natural language processing technique relies on the use of neural networks. In this review, we first discuss some recent papers that use one-hot encoding schemes in CRISPR-Cas9, followed by a brief overview of papers dealing with word embedding, and by the section presenting further sequence characteristics often used as explanatory features in ML and DL models.

It is worth noting that most of the sequence encoding schemes discussed in this section apply to both traditional machine learning and deep learning models. For example, the recent works of Lin *et al*., Wang *et al*., and Charlier *et al*. [56, 73–75] use the same sequence encoding schemes to provide the input of machine learning and deep learning models compared in these papers.

**Figure 3:**
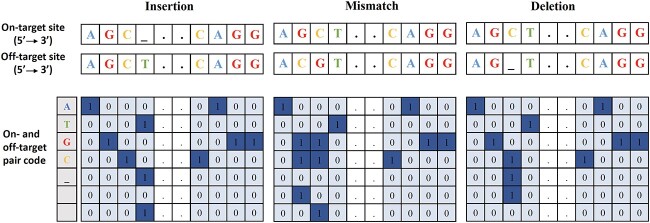
A novel effective sgRNA–DNA one-hot sequence encoding scheme used by Lin *et al*. [74]. A seven-bit encoding example is shown. Here, ‘_‘ symbol indicates the DNA or RNA bulge position. Each sgRNA–DNA sequence pair is encoded as a fixed-length seven-row matrix that includes a five-bit character channel (A, G, C, T, _) and a two-bit direction channel. The five-bit channel is used to encode the on- and off-target site nucleotides, whereas the direction channel is used to indicate the mismatch and indel locations.

In this paragraph, we present recent studies using novel one-hot encoding techniques in the field of genome editing. When applying a one-hot encoding, each sgRNA–DNA sequence pair of length $L$ can be encoded in a one-hot matrix of four rows and $L$ columns. Each row corresponds to the nucleotide type, i.e. A, C, G, and T (or U). Each base in the sgRNA and the target DNA is then encoded in the form of a one-hot vector according to a particular method. For example, Chuai *et al*. [71] proposed the deepCRISPR model for sgRNA on- and off-target prediction. DeepCRISPR relies on a deep convolutionary denoising neural network and one-hot data pre-processing. The nucleotide sequence is a 20-bp sgRNA sequence with an NGG PAM across the human genome. It is represented by four channels, (A, C, G, and T), and each epigenetic feature is considered as one channel. Thus, the encoded matrix used by Chuai *et al*. is of size $(4 + n) \times 23 $, where 4 corresponds to the number of channels and $n$ to the number of epigenetic features. Lin *et al*. [73] introduced a one-hot sequence encoding method that converts each sgRNA–DNA sequence pair into a matrix to be used as a convolutional input. In their encoding the four channels are used to represent both sgRNA and target DNA. Thus, each character in the sgRNA and target DNA sequences is represented by a single one-hot vector. Consequently, every sgRNA–DNA sequence pair is encoded in a matrix of size $4 \times 23$, where $23$ corresponds to the 3-bp PAM adjacent to the 20 bases. The use of such $4 \times 23$ input matrices allowed the authors to apply for the first time deep Feedforward Neural Networks (FNNs) and deep Convolutional Neural Networks (CNNs) for off-target prediction in CRISPR-Cas9 gene editing. Charlier *et al*. [75] described a different novel one-hot encoding method. Their main idea was to build a data encoding procedure that relies on a bijective mapping for sgRNA–DNA sequence pairs. It allows for encoding, and decoding, of the sgRNA–DNA sequence pairs without any information loss that can occur in the encoding scheme adopted in [73]. Specifically, Charlier *et al*. combined a $4 \times 23$ matrix used for sgRNA encoding and a $4 \times 23$ matrix used for DNA encoding, resulting in a $8 \times 23 $ matrix used as a convolutional input. The authors applied FNNs, CNNs, and Recurrent Neural Networks (RNNs) to generate accurate off-target predictions. Lin *et al*. [74] have recently introduced an encoding technique capable of incorporating in the input data base mismatch, missing base (RNA bulge or insertion), and extra-base (DNA bulge or deletion) information. Each sequence pair was considered as a fixed length vector with the following five-bit channel: (A, C, G, T, $\_$). Additionally, the authors introduced a two-bit direction channel that was used to identify the indel and mismatch directions. Thus, a combined seven-bit channel, encoded as seven one-hot encoded vectors, allowed them to take into account not only sgRNA–DNA sequence mismatches, but insertions and deletions as well. Precisely, Lin *et al*. [74] used a $7\times 23$ matrix encoding scheme (see Figure 3), where 23 is the length of the sgRNA–DNA sequence pairs. This encoding scheme is a perfect example of feature engineering, i.e. new feature construction process that is explicitly defined and manually or automatically applied. Feature engineering is common in machine learning. Moreover, some machine learning methods, e.g. Support Vector Machine (SVMs), incorporate feature engineering as part of their operation [76]. In the case of the sgRNA–DNA sequence encoding proposed by Lin *et al*. the created seven-bit-long features allow one to take into account all possible correspondences existing between the original sgRNA–DNA pairs of features taking the values (A, C, G, T, $\_$). This innovative encoding scheme was used with different deep learning models for off-target prediction on CIRCLE-Seq and GUIDE-Seq data sets, and demonstrated state-of-the-art prediction performance. Zhang *et al*. [77] designed an encoding scheme consisting of a matrix of size $20\times L$, with $L$ being the sequence length. In the encoding process, the authors used a four-bit channel (A, C, G, T) for sgRNA encoding, a four-bit channel (A, C, G, T) for DNA encoding, and a 12-bit channel to one-hot encode all possible mismatches. They regrouped the three corresponding matrices, resulting in a final matrix of size $20\times L$. This extended matrix was then used for data augmentation to reduce the class imbalance between off-targets and on-targets, while a CNN model was used for on-target activity prediction. Zhang *et al*. [78] proposed another encoding scheme with a similar objective to incorporate mismatch, DNA, and RNA bulge information into different off-target prediction models. The authors first considered a four-bit channel (A, C, G, T) and a one-hot vector encoding scheme. Furthermore, they used a two-bit channel to indicate a base deletion on RNA and DNA, and another one-bit function channel to indicate if the location is part of the guide sequence (0) or the PAM sequence (1). The encoded matrix was thus of size $7\times 23$. Finally, an ‘OR’ operation was carried out to indicate when two bases in a base pair were identical. Zhang *et al*. tested their encoding scheme with different FNN, CNN, and RNN models. They demonstrated performance on par with state-of-the-art. Overall, among the different one-hot encoding schemes found in the literature, the highest potential has been recently demonstrated by those relying on the use of indels.

The second common data pre-processing strategy used by several researchers is a Natural Language Processing (NLP) technique, called word embedding. The idea of applying word embedding on sgRNAs is that off-targets are encoded closer to each other in the vector space than are on-targets. Liu *et al*. [79] combined the word embedding with a transformer to convey sgRNA sequences to a deep neural network (DNN) model consisting of CNNs and FNNs. The authors demonstrated that the word embedding approach had similar predictive performance as the latest one-hot encoding-based deep learning models. Later on, Liu *et al*. [80] proposed to use a trained unsupervised learning algorithm, GloVe [81], designed to aggregate word-to-word occurrence statistics outputting linear substructures of the word vector space. The authors applied GloVe to convert sgRNA sequences into substructures of the word vector space. They forwarded the sgRNA word vectors to a bidirectional LSTM and a CNN with five convolutional layers to predict the sgRNA off-target propensity, and demonstrated state-of-the-art predictive performance of their models. Zhang *et al*. [82] proposed to label-encode and word-embed sgRNA sequences. Each sgRNA sequence was transformed into a numerical vector using the Tokenizer module from the Keras library [83]. The encoded sequences were then passed to pooling and convolutional layers, and to three convolutional layers to obtain sgRNA cleavage efficiency predictions. Using word-embedding techniques from NLP is fairly recent, but we are confident that it opens new perspectives for future work in the field of genome editing.

One-hot encoding and word embedding are not the only ways to represent a sequence in machine learning models. The majority of conventional machine learning (ML) models used in CRISPR-Cas9 consider additional sequence features such as position-specific features, different counts, and structural/thermodynamic characteristics to best capture sequence information (see Table 2). In contrast to conventional machine learning models, deep learning (DL) models can automatically learn the sequence features by generating new internal features that are crucial for accurate outcome predictions (see, for example, the convolutional deep learning models used by Lin *et al*. [74] and Shrawgi *et al*. [84], and the discussion therein). Here, we briefly recall some important works in the field and highlight sequence features that helped to boost the performance of the related machine learning models. Doench *et al*. [54], Xu *et al*.[61], and Peng *et al*. [85] identified several sgRNA and target DNA features allowing one to improve the prediction results, including position-specific nucleotide composition for individual (order 1) nucleotides (A/C/T/G) and pairwise (order 2) nucleotides (AA/AT/AG/...) in 30mer sequences (i.e. the 20mer guide plus context on either side) and GC counts for each guide sequence. Afterwards, Doench *et al*. [59] used thermodynamic features and position-independent features in addition to previously considered position-specific features and the GC counts (Doench *et al*. [54]). Position-independent features included individual nucleotide counts (order 1) and adjacent pairwise nucleotide counts (order 2), ignoring their position in the sgRNA. Thermodynamic features were computed from the melting temperatures of the DNA version of the RNA guide sequence, or portions thereof, using the Biopython Tm_staluc function [59]. Rahman *et al*. [86] considered position-specific features, position-independent features, as well as sgRNAs secondary structures to create additional features for their ML models. The structural features used by Rahman *et al*., which are also known as thermodynamic properties, included Minimum Free Energy, the most favorable thermodynamic RNA–RNA interaction energy, local pair probabilities and specific sgRNA heat parameters. In their CRISPRO experiments, Schoonenberg *et al*. [87] considered several categorical and numerical features. The categorical features used in this work include targeted amino acids 1 and 2, domain occupancy status (InterPro), exon multiple of 3, the ability of targeted transcript to escape nonsense-mediated decay, single nucleotide and dinucleotide positional identities within sgRNA spacer, and orientation of sgRNA relative to gene. All categorical features were one-hot encoded. Numerical features considered by Schoonenberg *et al*. include the PROVEAN (Protein Variation Effect Analyzer) deletion score of the targeted amino acids 1 and 2, position in the gene, predicted disorder score of amino acids 1 and 2, GC content of the 20-mer guide, length of the targeted exon, and off-target score of the gRNA. Chen *et al*. [88] used two categorical features, i.e. the mismatched bases on both the gRNA (called ‘Ref allele’) and donor DNA (called ‘Alt allele’), and one numerical feature, i.e. mismatch position relative to the gRNA. These features were then converted into binary vectors using one-hot encoding and employed as input of several traditional ML models tested by the authors. Rafid *et al*. [89] proposed an accurate SVM-based tool using sequence-based features only. The authors experimented with three types of features, including position-independent features, position-specific features, and n-gapped dinucleotides (nGD). The nGD features count the number of times that two given nucleotides appear at a certain distance in a sgRNA sequence. Dhanjal *et al*. [90] explored 11 categories of sequence features that possibly govern the specificity of sgRNAs. The main finding of their work consists in the identification of the four most important sequence features, including accessibility of target sequence in the genome, mismatch count between the off-target and target sequences, position-specific occurrence of nucleotides in the spacer and regions flanking it on both sides, and, finally, GC count in the target and off-target sequences. Hiranniramol *et al*. [58] used sequence and structural features of the most and the least potent sgRNAs to train their sgDesigner model. Significance levels for numerical features were computed using Student's t-test, and for binary features using the $\chi ^2$ test. The authors considered only the high- and low-efficiency sgRNAs groups to emphasize the most predictive features affecting sgRNA efficiency. He *et al*. [91] demonstrated that sequence-specific sgRNA activity, frameshift probability and amino acid features could significantly improve the selection of efficient sgRNAs in protein knockouts. They highlighted the importance of amino acid sensitivity as one of the critical factors that govern the efficiency prediction, in addition to the use of effective sequence models that predict sgRNA activity.

**Table 2:** A summary of studies applying traditional machine learning methods for on and off-target prediction in CRISPR/Cas9.

Study	Year	Target	ML model(s)	Encoding	Data	Link for data/	Prediction metric/
		prediction	used			software	results
Wang *et al*.	2014	On-target	SVM	One-hot +	A library of 73 000 sgRNAs	https://www.ncbi.	log2 fold
[52]				GC content	was used to generate	nlm.nih.gov/	change
					knockout collections	pmc/articles/	estimations
					for two human cell lines	PMC3972032	
Doench *et al*.	2014	On-target	SVM and	One-hot +	1831 gRNAs targeting	http://portals.	AUROC: 0.8
[54]			Logistic	GC counts +	three human genes and	broadinstitute.	
			regression	position-specific	six mouse genes were used	org/gpp/public/	
				features	to generate screening	analysis-tools/	
					data; Doench V1	sgrna-design	
Xu *et al*.	2015	On-target	Logistic	One-hot +	Wang [52],	http://crispr.	AUROC: 0.73
[61]			regression	GC counts +	Koike-Yusa [53],	dfci.harvard.	
				position-specific	Shalem [105],	edu/SSC	
				features	Zhou [106]		
					Gilbert [107]		
					Konermann [108]		
Fusi *et al*.	2015	On-target	SVM,	One-hot +	Wang ribosomal,	http://research.	Spearman: 0.52
[100]			L1 regression,	GC counts +	Wang non-	microsoft.com/	AUROC: 0.75
			L2 regression,	position-specific	ribosomal [52],	enus/projects/	
			RF regression,	features	Koike-Yusa [53],	azimuth	
			SVM+logistic		Doench V1 [54]		
			regression,				
			L1 logistic				
			regression,				
			linear				
			regression,				
			GBRT				
Doench *et al*.	2016	Off-target	Boosted RT,	One-hot +	2549 unique guides	http://www.	Spearman: 0.54
[59]		and	L1 regression,	GC counts +	were used to	broadinstitute.	for on-target
		on-target	L2 regression,	position-specific	generate Doench V2	org/rnai/public/	AUROC: 0.8
			SVM+logistic	features +	data set	analysistools/	for off-target
			regression,	position-independent		sgrna-design,	
			RF, linear	features +		http://research.	
			regression	thermodynamic		microsoft.com/	
				features		enus/projects/	
						azimuth	
Rahman *et al*.	2017	On-target	CRISPRpred	One-hot +	Doench V1 [54]	https://github.	AUROC: 0.85
[86]			(SVM, RF,	position-specific		com/khaled-buet/	AUPRC: 0.56
			linear	features +		CRISPRpred	MCC: 0.4
			regression)	position-independent			
				features +			
				structural/			
				thermodynamic			
				features			
Abadi *et al*.	2017	Off-target	CRISTA	GC content +	GUIDE-Seq [43, 47],	http://journals.plos.	Spearman: 0.81
[46]			(CRISPR	sgRNA secondary	HTGTS [48],	org/ploscompbiol/	AUROC: 0.96
			Target	structure features	BLESS [49, 50]	article?id=10.1371/	AUPRC: 0.96
			Assessment			journal.pcbi.1005807	$R^2$ = 0.8.
			using RF				
			regression)				
Peng *et al*.	2018	Off-target	Ensemble SVM	One-hot +	CRISPOR [60]	https://github.	AUROC: 0.99
[85]				GC content +		com/penn-hui/	AUPRC: 0.45
				position-specific		OfftargetPredict,	
				features		Cas-OFFinder [109]	
Schoonenberg	2018	On-target	CRISPRO	One-hot +	Doench V2 [59],	http://gitlab.	Spearman: 0.57
*et al*. [87]			(GBDT,	GC content +	Munoz [70],	com/bauerlab/	
			Ridge, RF,	position-specific	Donovan [110],	crispro	
			Lasso, SVM)	features	Brenan [111]		
Listgarten	2018	Off-target	Elevation	One-hot	GUIDE-Seq [43],	http://research.	AUROC: 0.98
*et al*. [112]			(Boosted		Doench V2 [59],	microsoft.com/	
			regression		CRISPOR [60]	en-us/projects/	
			Trees,			crispr	
			L1 regression,				
			Naïve Bayes)				
Chen *et al*.	2019	Off-target	Logistic	One-hot +	Unpublished data of	https://github.	Accuracy: 94$\%$.
[88]			regression,	Ref allele +	Sharon *et al*. were	com/elizapan	
			SVM, RF,	Alt allele +	used to generate	dabella/CRISPEY	
			NN	mismatch position	CRISPEY (Cas9-Retron	_ML_Public	
				in guide	precISe Parallel Editing		
				features	via homologY) data		
					consisted of 23,936		
					samples (18,717 training		
					set and 4,680 testing set)		
Zhang *et al*.	2019	Off-target	Ensemble	One-hot +	GUIDE-Seq [43],	https://github.com/	AUROC: 0.938
[95]			learning	GC counts +	CRISPOR [60]	Alexzsx/CRISPR	AUPRC: 0.299
			framework	position-specific			
			of scoring	features			
			features				
			using				
			AdaBoost				
Lazzarotto	2020	Off-target	CHANGE-seq	One-hot +	High-throughput	https://github.	AUROC: 0.995
*et al*. [51]			(GTB)	sequence	sequencing data	com/tsailabSJ/	AUPRC:0.881
				features	generated	changeseq,	
					(CHANGE-seq),	Cas-OFFinder [109],	
					GUIDE-Seq [43],	DeepTools [113]	
					CIRCLE-seq [44]		
Rafid *et al*.	2020	On-target	CRISPR	Position-	CRISPOR [60],	https://github.	Spearman:0.829
[89]			pred(SEQ),	independent +	DeepHF [56]	com/Rafid013/	AUROC: 0.893
			(SVM)	position-specific		CRISPRpredSEQ	
				features +			
				n-gapped			
				di-nucleotide			
He *et al*.	2020	On-target	GuidePro	Sequence-specific	Doench V1 [54],	https://bioinfor	Spearman:0.523
[91]			(two-layer	features	Doench V2 [59],	matics.mdanderson.	
			ensemble,		CRISPOR [60],	org/apps/GuidePro,	
			SVM and RF)		Munoz [70],	https://github.	
					Schoonenberg [87],	com/MDhewei/	
					Aguirre [114],	GuidePro,	
					Evers [115],	inDelphi [116],	
					Bertomeu [117],	Lindel [118],	
						FORECasT [119]	
Wang *et al*.	2020	On-target	GNL-Scorer	One-hot +	10 public data sets:	https://github.	Spearman: 0.502
[101]			Eight models;	GC count +	Doench V1 [54],	com/TerminatorJ/	
			(GBRT, DT,	position-	Doench V2 [59],	GNL_Scorer	
			linear	independent +	HCT116 [62],		
			regression,	position-	Hela [62],		
			L2 regression,	dependent	Z_fish MM [63],		
			L1 regression,	features +	Z_fish VZ [64],		
			BRR, RF, NN)	thermodynamic	Z_fish GZ [65],		
				features	Drosophila [66],		
					HEK293T [67],		
					Ciona [68]		
Dhanjal *et al*.	2020	Off-target	L1 logistic	One-hot +	CIRCLE-seq [44],	http://web.iitd.	Accuracy: 91.49$\%$
[90]			regression,	GC content +	CRISPcut [120]	ac.in/crispcut/	AUROC: 0.97
			L2 logistic	position-specific		off-targets	
			regression,	features			
			RF, xgboost				
X. Liu *et al*.	2020	Off-target	SeqCor (open-	A general-	DeepCRISPR [71]	https://github.	Spearman: 0.4
[104]		and	source software	purpose hash		com/wangyi-fud	for off-targets,
		on-target	bundle to correct	function		an/SeqCor	and 0.369 for on-targets
			the experimental				
			data using				
			random				
			forest-based)				
Hiranniramol	2020	On-target	sgDesigner	GC content +	Wang [52],	https://github.	Spearman: 0.75
*et al*. [58]			(stacking SVM	structural	Koike-Yusa [53],	com/wang-lab/	AUROC: 0.934
			and XGBoost	features	Doench V1 [54],	sgDesigner,	Accuracy: 86.3$\%$
			using logistic		Chari [67],	RNAfold [121]	
			regression)		Shalem [105]		
Konstantakos	2022	On-target	CRISPRedict	Overall and	Koike-Yusa [53],	https://github.	Spearman: 0.380
*et al*. [102]			(linear	position-specific	DeepSpCas9 [57],	com/VKonstant	for U6 data sets,
			regression,	nucleotide	CRISPOR [60],	akos/CRISPRedict,	and 0.355 for
			binomial	composition +	Labuhn [71, 122],	http://www.	T7 data sets,
			regression,	structural		crispredict.org	nDCG: 0.805 for
			logistic	properties of			U6 data sets,
			regression)	sRNAs			and 0.554 for
							T7 data sets
Zarate *et al*.	2022	On-target	BoostMEC	GC content +	DeepSpCas9 [57],	https://github.	Spearman: 0.78
[103]			(Boosting	position-specific	CRISPRon [123]	com/oazarate/	
			Model for	features +		BoostMEC	
			Efficient	thermodynamic			
			CRISPR)	features			

RF: Random forest, GBRT: Gradient-boosted regression tree, SVM: Support Vector machine, MCC: Matthews correlation coefficient, GB: Gradient boosting, NN: Neural networks, KNN: K-nearest neighbors, GTB: Gradient tree boosting, DT: Decision tree, BRR: Bayesian ridge regression, nDCG: Normalized discounted cumulative gain.

In conclusion, we need to point out that feature comparison between different experiential CRISPR-Cas9 data sets uncover substantial discordance and that further research is warranted to identify the most significant generic predictors (i.e. explanatory features) in case of both guide efficiency (i.e. on-target activity) and guide specificity (i.e. off-target effects).

## TRADITIONAL MACHINE LEARNING MODELS AND THEIR APPLICATIONS IN CRISPR/Cas9

In this section, we present different conventional machine learning models for on- and off-target prediction found in the genome editing literature related to CRISPR/Cas9. The presentation is organized based on the target categories: (1) off-target prediction only, (2) on-target prediction only, and finally (3) both on- and off-target prediction. Within each category, the works follow chronological order.

First, we discuss papers dealing with off-target activity prediction. Abadi *et al*. [46] proposed the CRISPR Target Assessment (CRISTA) algorithm that relies on a random forest ensemble machine learning framework to determine the propensity of a genomic site to be cleaved by a given sgRNA. The authors determined that the system attributes representing spatial structure and rigidity of the entire genomic site, as well as those related to the PAM region have the main impact on the prediction capabilities. Peng *et al*. [85] were among the first authors to capitalize on the recent advances in CRISPR/Cas9 data availability. They experimented with two positive sample sets, comprising both on- and off-targets. The first of them contains 215 sequence pairs related to 29 sgRNAs’ on-target and off-target editing sites. The second data set includes 527 sequence pairs obtained using high-throughput sequencing techniques—Digenome-seq [25], GUIDE-seq [43], HTGTS [48], CIRCLE-seq [44], and multiplex Digenome-seq [92]. The authors randomly under-sampled the data to compensate for the class imbalance between on- and off-targets. Then, they trained an ensemble SVM classifier to detect the off-target sites. The authors demonstrated the ability of their model to outperform state-of-the-art predictive methods by aggregating larger numbers of sgRNA–DNA sequence pairs. The work of Peng *et al*. opened new directions for data aggregation in the field of genome editing. Chen *et al*. [88] generated the CRISPEY data set consisting of 23 936 samples, each of which contains a 20-nucleotide gRNA sequence and a 100-basepair donor DNA sequence. In this data set, 306 samples are labeled as effect samples and 23 630 samples are labeled as no-effect samples. To predict eventual off-targets, the authors applied three conventional machine learning algorithms including logistic regression, SVM [93], and random forest [94], as well as a simple DNN. The SVM model with a recall rate of 64% and the logistic regression with an accuracy of 94% provided the best prediction results overall. Zhang *et al*. [95] explored the ensemble learning potential for off-target prediction by synergizing multiple tools. The input of their ensemble learning model included five scores calculated by the following scoring methods: CCTop [27], MIT Website [28], CFD [54], MIT [60], and Cropit [96], as well as evolutionary conservation data and Chromatin state segmentation data. The authors considered an imbalanced data set containing 25 332 putative off-target DNA sequences, with 152 verified positive off-targets. They compared the five following machine learning algorithms—AdaBoost [97], random forest, a multi-layer perceptron [98], SVM, and decision trees [99]. In their experiments, Zhang *et al*. demonstrated that the ensemble-based AdaBoost algorithm was able to outperform the other predictive algorithms in terms of the area under the precision recall curve (AUPRC) and the area under the receiver operating characteristic curve (AUROC) metrics. Lazzarotto *et al*. [51] proposed an approach targeting the fast pace of changes in genome editing with a scalable, automatable tagmentation-based model for estimating the genome-wide Cas9 *in vitro* activity. Their CHANGE-Seq model was designed to better understand the specificity of genome editors. In their experiments, Lazzarotto *et al*. used the encoded one-dimensional vectors to train a gradient tree boosting model to predict off-target activities. The authors highlighted the importance of the protospacer and the PAM position to ensure accurate off-target predictions. Moreover, they showed that CHANGE-Seq generally outperforms the well-known GUIDE-seq [43] and CIRCLE-seq [44] models.

Second, we present a summary of recent papers addressing the problem of machine learning prediction of on-target activities. Wang *et al*. [52] were among the first authors to use SVMs to predict sgRNA efficacy. The authors used log2 fold change of sgRNAs targeting ribosomal protein genes as their efficacy indicator. Precisely, the log2 fold change was applied to build a binary classification, where ribosomal protein gene-targeting sgRNAs were designated either as weak or as strong. Doench *et al*. [54] trained a logistic regression classifier to differentiate the highest activity quintile of sgRNAs from their lowest activity quintile. The authors used sequence features from nine mouse and human genes with cross-validation to ensure the generalization across genes. Xu *et al*. [61] applied a regularized regression technique that linearly combines the penalties of the Lasso and Ridge methods, and Elastic-Net, to predict sgRNA efficiency in CRISPR/Cas9 knockout experiments. The authors demonstrated that Elastic-Net outperforms existing models on different independent data sets. Fusi *et al*. [100] investigated how to achieve the best optimal predicative performance in CRISPR/Cas9 gene editing. The authors relied on two different primary data sets composed of mouse and human genes. They built and trained five traditional machine learning classifiers to predict the knockout efficacy, and observed that the gradient-boosted regression trees yielded the best performance overall. Rahman *et al*. [86] introduced the CRISPRpred model aiming at providing accurate *in silico* predictions of sgRNA on-target activity. CRISPRpred is capable to extract relevant features in order to use them in an SVM-based machine learning framework. The work of Rahman *et al*. emphasizes the importance of feature engineering in boosting the predictive performance of sgRNA on-target prediction models. Furthermore, Rafid *et al*. [89] demonstrated the importance of feature engineering and data pre-processing to ensure effective sgRNA on-target activity prediction. The authors proposed a novel SVM-based machine learning tool, named CRISPRpred(SEQ), which is capable to challenge the effective DeepCRISPR model [71] based on deep learning. The authors demonstrated that due to designing better explanatory features, CRISPRpred(SEQ), which used a simpler model architecture, was able to outperform DeepCRISPR in three out of four cell lines. Wang *et al*. [101] proposed a novel methodology targeting cross-species generalization of on-target activities. The authors developed the GNL-Scorer software computing two cross-species generalization scores, GNL and GNL-Human. GNL-Scorer also combines different data sets, features and models for sgRNA activity prediction, agnostic to the species. The authors claimed that GNL-Scorer facilitates the current *in silico* design of sgRNAs. Konstantakos *et al*. [102] introduced a new interpretable gRNA efficiency prediction model and the related web tool, called CRISPRedict, including various regression and classification models for gRNA scoring. This web tool offers accurate efficiency predictions under different experimental conditions (e.g. U6/T7 transcription) and the related visualizations facilitating the explanation of the obtained results. As explanatory features, the authors considered overall and position-specific nucleotide composition, as well as variables reflecting the structural properties of gRNAs. They conducted a multi-step feature selection analysis to infer a minimal relevant feature subset. Then, they used a binomial and a linear regression models to predict the percentage of successful edits for the U6 and T7 variants, and trained two logistic regression models by labeling the top 20% and the bottom 20% of gRNAs as efficient and inefficient, respectively. Konstantakos *et al*. evaluated the performance of CRISPRedict, comparing it with state-of-the-art gRNAs design tools, including some deep learning models, and concluded that despite its simplicity, CRISPRedict provides interpretable efficiency predictions with comparable performance. Zarate *et al*. [103] developed a new machine learning model, called BoostMEC (Boosting Model for Efficient CRISPR), to predict CRISPR-Cas9 editing efficiency. BoostMEC is based on a gradient boosting technique and LightGBM (Light Gradient-Boosting Machine). The LightGBM hyperparameters were tuned using tenfold cross-validation and Bayesian hyperparameter optimization. The authors compared BoostMec with 10 state-of-the-art on-target prediction models on 13 benchmark data sets. They concluded that BoostMEC, which relies on direct and derived sgRNA features and traditional machine learning, has an advantage over state-of-the-art prediction models based on deep learning because of its ability to produce more interpretable feature insights and predictions.

Finally, we discuss papers addressing the problem of both on- and off-target activity prediction by means of conventional machine learning models. Doench *et al*. [59] designed and tested their novel sgRNA design rules to create human and mouse genome-wide libraries and carry out the corresponding positive and negative selection screens. The authors proposed a new metric to predict off-target sites, and designed optimized sgRNA libraries with maximized on-target activity and minimized off-target effects. In order to identify an optimal classifier, they compared the performance of eight conventional machine learning models, including linear regression, L1-regularized linear regression, L2-regularized linear regression, a hybrid SVM plus logistic regression, random forest, gradient-boosted regression trees, L1 logistic regression (a classifier), and SVM (with linear kernel with default L2 regularization). Liu *et al*. [104] proposed an open-source software, called SeqCor, which relies on the application of the random forest algorithm to extract sequence features that influence gRNA knockout efficiency as well as gRNA off-target activity at specific sites. The aim of their work was to facilitate the extraction of the sequence features and to minimize possible bias effects that may be present in a library used in CRISPR/Cas9-based screening.

Although the use of traditional machine learning algorithms, whose main advantages are their relative simplicity and fast training, led to some impressive on- and off-target activity prediction results, recent studies conducted using deep learning methods (see the next section) often demonstrated a superior performance. We are convinced that, in general, deep learning models are better suited for both on- and off-target activity prediction than conventional machine learning models, since modern CRISPR/Cas9 data sets contain hundreds of thousands, and sometimes millions, of samples, and state-of-the-art deep learning algorithms can be effectively used on such huge volumes of data encompassing complex non-linear patterns. However, for benchmark purpose, the results provided by deep learning algorithms should be always compared with those yielded by some well-performing traditional machine learning methods such as SVM, random forest and XGBoost, as well as their ensemble frameworks, which are capable of increasing the accuracy of individual methods.

Table 2 reports the main traditional machine learning classifiers and regressors used for on- and off-target prediction in CRISPR/Cas9.

## A BRIEF REVIEW OF DEEP NEURAL NETWORKS

Deep learning applications across all research fields have recently gained popularity due to easier access to data, boosted computing power, and recent theoretical progress in supervised learning. DNNs are at the core of deep learning. They are capable of learning complex patterns from the data using multiple layers of interconnected neurons. Nonetheless, their training and optimization are still very challenging problems. This section is divided into two parts. First, we present the main properties of existing deep learning network architectures. Second, we discuss their applications in CRISPR/Cas9.

**Figure 4:**
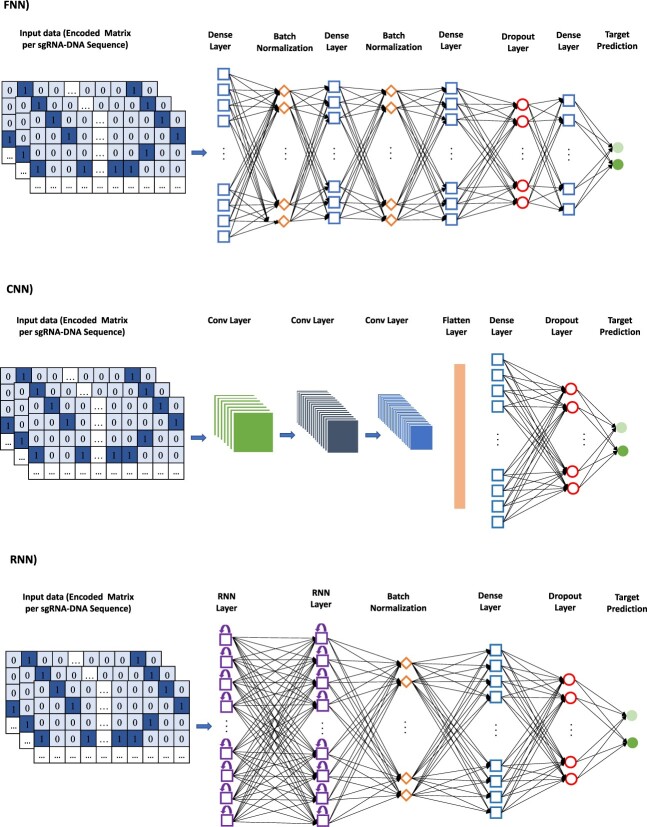
Typical FNN, CNN, and RNN architectures used to predict on-and off-targets in CRISPR/Cas9. For each network, the encoded matrix containing the sgRNA–DNA sequence pair information is used as input (for more details, see Charlier *et al*. [75]).

In this section, we describe succinctly the three main types of DNNs used for on- and off-target activity prediction in CRISPR/Cas9. They are FNNs, CNNs, and RNNs. Figure 4 illustrates three typical deep learning network architectures used in CRISPR/Cas9. Finally, we present some popular activation functions of neurons used in deep learning models.

FNNs are one of the most popular DNN models [76]. In a standard FNN architecture, the information always moves forward between different layers of interconnected neurons. The two main types of FNN are as follows: a single-layer FNN and a multi-layer FNN. In a single-layer FNN, the input layer, i.e. the first layer of neurons receiving the data as input, is directly fully connected to the output layer. The output layer is the last layer outputting the predictions. In a multi-layer FNN, the input layer and the output layer are fully connected to hidden layers. Thus, a multi-layer FNN has at least three layers of neurons. Figure 4 presents a typical multi-layer FNN architecture used for off-target predictions in CRISPR/Cas9 (for more details, see [75]).

CNNs, introduced by Lecun *et al*. [124], rely, as FNNs, on fully connected layers, but also include convolutional and pooling layers. A convolutional layer consists of a collection of convolutional filters used to extract spatial features from the input image. Within the convolutional layer, different filters, or kernels, can be applied to process the data and generate feature maps. These feature maps help the neural network to better regress or classify the input data. Following the convolutional layers, hidden fully connected layers are often used to further improve the predictive performance of a CNN. An example of a CNN architecture used for off-target prediction in CRISPR/Cas9 is presented in Figure 4 (for more details, see [75]).

RNNs are another type of neural networks that can be used effectively for on- and off-target prediction in CRISPR/Cas9. In contrast to FNNs and CNNs, the information in RNNs does not always move forward; it can also move backward. The aim of RNNs is to replicate a memory process through a recurrent learning mechanism. RNNs aggregate information of the past inputs and that of the current input in order to produce the current output. An example of an RNN architecture used for off-target prediction in CRISPR/Cas9 is presented in Figure 4 (see also [75]). Two popular types of RNNs are Long Short-Term Memory (LSTM) models, which are designed to learn order dependence in sequence prediction problems [125, 126] and Gated Recurrent Unit (GRU) models, which use a similar to an LSTM prediction mechanism, but require less memory and are usually faster than LSTMs as they have no output gate [127]. Both LSTM and GRU model architectures have the ability to forget the information using a forget gate. One of the advantages of LSTMs is that they are able to overcome the vanishing gradient problem that occurs while training networks with backpropagation and gradient-based learning methods, preventing undesirable weight updates in the RNN. For the input sequence $<x_1, x_2, \ldots , x_T>$, the key mathematical equations of the forward pass of an LSTM unit are as follows [76]: 


(1) \begin{align*} f_t &= \sigma_g (W_f x_t + U_f h_{t-1}+b_f),\\ \nonumber i_t &= \sigma_g (W_i x_t + U_i h_{t-1}+b_i),\\ \nonumber o_t &= \sigma_g (W_o x_t + U_o h_{t-1}+b_o),\\ \nonumber c_t &= f_t \circ c_{t-1} + i_t \circ \sigma_c (W_c x_t + U_c h_{t-1}+b_c),\\ \nonumber h_t &= o_t \circ \sigma_h (c_t), \end{align*}

where $\circ $ denotes the Hadamard product, $x_t$ is the unit’s input at time $t$, $h_t$ is the corresponding unit’s output, $c_t$ is the hidden unit’s memory, and $i_t$, $f_t$ and $o_t$ are, respectively, the activation vectors of the input gate, of the forget gate, and of the output gate. The variables $W$, $U$ and $b$ are, respectively, the weight matrices and the bias parameter, and $\sigma _g$ denotes a sigmoid function. Finally, both $\sigma _c$ and $\sigma _h$ denote hyperbolic tangent functions.

For all deep learning network architectures presented here, the neurons rely on an activation function that is used to determine whether a given neuron should be activated or not. Thus, the activation function being used determines whether the neuron’s contribution to the network is important or not in the prediction process. An activation function allows the model to transform the weighted sum of the neuron’s input signals into an output signal. Table 3 summarizes the most common activation functions used with artificial neural networks.

**Table 3:** Activation functions commonly used with artificial neural networks.

Name of the function	Formula
ReLU	$\sigma (x) = \max (0, x)$
Leaky ReLU	$\sigma (x) = \max (\alpha x, x)$
Randomized leaky ReLU (RReLU)	$\sigma (x) = \max (0, x) + \alpha \times \min (0, x)$
Parametric leaky ReLU (PReLU)	$\sigma (x) = \max (0, x) - \alpha \times \max (0, -x) $
Scaled exponential Linear Units (SeLU)	$$\sigma (x)= \lambda \begin{cases} x, \kern22pt x> 0\\ \alpha \ e^x - \alpha , x\leq 0 \end{cases} $$
Logistic (Sigmoid)	$\sigma (x) = \dfrac{1}{1+e^{-x}}$
Hyperbolic Tangent (Tanh)	$\sigma (x) = \dfrac{e^{x} - e^{-x}}{e^{x} + e^{-x}}$
Softmax	$\sigma(x)_i = \dfrac{e^{x_i}}{\sum _{j=1} ^K e^{x_j}},\ \text{where }K\text{ is the number of classes}$

DNNs, by their nature, have a large number of parameters to fine-tune during their training. Special attention must be given to the problem of overfitting in the model’s training. Overfitting occurs when the model learns patterns only present in the training set and cannot generalize its predictive performance on the test set. Different techniques exist to limit the overfitting while training DNNs. The most important of them are early-stopping, network-reduction, regularization, and dropout [76, 128]. We invite the reader to consult the aforementioned literature for more details.

## DEEP LEARNING MODELS AND THEIR APPLICATIONS IN CRISPR/Cas9

This section includes four thematic subsections. First, we discuss the studies emphasizing the use of novel sequence encoding strategies along with deep learning models. Second, we present works using different feature engineering approaches. Third, we highlight the papers applying class rebalancing techniques prior to carrying out deep learning algorithms. Forth, we describe some recent works relying on the use of attention mechanism. Within each category, the works follow the off-target, on-target and both on- and off-target prediction order.

### Models relying on novel sequence encoding strategies

As regards the off-target prediction, Lin *et al*. [73] proposed a novel sequence encoding scheme based on the use of DNNs. They investigated several network architectures, including CNNs and FNNs. In their experiments, the authors used publicly available CRISPOR (18 236 samples) and GUIDE-Seq (430 samples) data sets. The transfer learning strategy was applied to obtain the off-target predictions for a much smaller GUIDE-Seq data set as the model trained on the CRISPOR data set was used to predict the GUIDE-Seq off-targets. Despite the fact that the encoding strategy proposed by Lin *et al*. [73] could lead to some information loss as the $4 \times 23$ matrix considered by the authors cannot fully represent the unique match-mismatch information of a given sgRNA–DNA sequence pair (it was then corrected by Lin *et al*. [74], who considered a loss-free model based on a $7 \times 23$ matrix encoding), very encouraging AUCROC results were obtained by these authors (i.e. an area under the curve values up to 0.972 were generated). The main conclusion of this work was that CNN and FNN deep learning networks steadily outperform state-of-the-art off-target scoring prediction methods (i.e. CFD, MIT, CROP-IT, and CCTOP) as well as some traditional machine learning classifiers (i.e. random forest, gradient boosting trees, and logistic regression). Afterwards, Lin *et al*. [74] proposed an original one-hot sequence encoding scheme (see Figure 3) and an effective CRISPR-Net model, where a Long-term Recurrent Convolutional Neural Network (LRCN) was playing the role of a feature extractor. The authors noticed that convolutional layers of LRCN were able to discover useful features from sequences directly and independently, preventing possible biases introduced by hand-crafted sequence features. The convolutional kernels within the LRCN were replaced by an inception module and a bidirectional LSTM was used for scoring the off-target activity of each potential sgRNA-target. Charlier *et al*. [75] proposed a novel sgRNA–DNA sequence encoding technique, which was applied in a deep learning off-target prediction framework. The loss-free encoding model introduced by the authors relied on $8 \times 23$ matrix representations. Charlier *et al*. compared the prediction performance of different FNN, CNN, and RNN network architectures (see Figure 4), as well as of several machine learning classifiers (i.e. random forest, naive Bayes, and logistic regression). The predictions were performed on two well-known gene editing data sets, CRISPOR and GUIDE-Seq, which were previously considered by Lin *et al*. [73]. The transfer learning approach was used as well to predict the off-targets on the much smaller GUIDE-Seq data set. The proposed prediction framework led to more accurate off-target prediction results, compared with those obtained by Lin *et al*. [73], yielding an improvement of the AUCROC metric up to 35%.

Regarding the on-target prediction, Xue *et al*. [129] introduced DeepCas9, an effective deep-learning framework based on CNNs. The authors proposed a network architecture to automatically learn the sequence determinants, conducting their experiments with 10 CRISPR/Cas9 data sets of different sizes. Xue *et al*. demonstrated that DeepCas9 is capable of outperforming some traditional machine learning methods such as random forest and logistic regression in terms of on-target activity prediction. In their timely review, Konstantakos *et al*. [37] evaluated 11 tools for gRNA efficiency prediction, which were applied to analyze six benchmark data sets. The evaluated tools included 10 deep learning models and one conventional machine learning model, called Azimuth 2.0 [59] and based on gradient-boosted regression trees. Most of the considered deep learning models represented the target sequence by means of different one-hot encoding strategies, while a few of them captured the epigenetic features as well. Comparison of gRNA efficiency prediction was carried out using the Spearman correlation. The authors observed that the correlation between the predicted and the true efficiency varied a lot, depending on the data set being analyzed and the model being used. Overall, the DeepHF [56] and DeepSpCas9 [57] models were consistently among the top performers along with the Average prediction strategy that consisted of the mean of the DeepCRISPR [71], DeepCas9 [129], DeepSpCas9 [57], and Azimuth 2.0 [59] predictions. Konstantakos *et al*. pointed out that simpler machine learning tools can sometimes outperform their much more sophisticated deep learning counterparts, and that large data sets that benefit from unbiased experimental measurements play a crucial role in training a generalizable model such as DeepHF or DeepSpCas9.

Concerning the prediction of both on- and off-targets, we need to mention the work of Chuai *et al*. [71], who were among the first to tackle the problem of sgRNA–DNA sequence encoding adapted to the input of deep learning models. The authors implemented a deep learning framework, called DeepCRISPR, predicting simultaneously the sgRNA on-target knockout efficacy and the off-target cleavage. An original one-hot encoding strategy used by the authors consisted of a four-channel-based sgRNA–DNA sequence encoding and epigenetic feature encoding in which each feature was considered as an independent channel. Chuai *et al*. introduced an unsupervised representation learning strategy to train a Deep Convolutional Denoising Neural Network (DCDNN) auto-encoder to learn the underlying representation of sgRNAs. The unsupervised deep representation learning approach was then used to transfer the encoded data to a hybrid DNN. The proposed network included a softmax activation function and an identity function for the classification and regression tasks, respectively. DeepCRISPR provided good prediction results in terms of both AUPRC and AUCROC, compared with the CFD prediction method [54].

Most of the aforementioned studies demonstrated impressive prediction results using novel sgRNA–DNA one-hot sequence encoding schemes combined with deep learning models. We think, however, that further prediction improvements will not anymore result from the use of sophisticated data encoding schemes, nor from the models complexity, but rather from an effective use of additional biological or physical features as well as from the application of the feature engineering and class rebalancing techniques.

### Models relying on feature engineering

In addition to conventional nucleotide sequence data, some plausible biological and physical features, such as gene melting temperature, molecular weight or microhomology features, can be also used as input of CRISPR/Cas9 predictive models. Moreover, some new informative features can be created, or reengineered, from the existing ones. Feature engineering has proven to be an important milestone when developing and optimizing predictive models [76].

As regards the off-target prediction, Liu *et al*. [80] introduced a deep learning architecture, called CnnCrispr, to predict the off-target propensity of sgRNAs at specific DNA fragments. The approach proposed by these researchers relies on the GloVe embedding model [81] to extract the global statistical information from genes. The constructed word vector matrix was then embedded into the considered deep learning model including a bidirectional LSTM and a CNN. The authors demonstrated that the proposed approach outperforms state-of-the-art classification and regression algorithms. Stortz *et al*. [130] introduced the piCRISPR deep learning model intended for off-target prediction using physically informed features. The authors designed four different feature encoding schemes to incorporate the following physically informed features: the target-guide encoding, the target-mismatch encoding, the target-mismatch-type encoding, and the target-OR-guide encoding. Moreover, they assessed the feature importance using the model-agnostic SHAP (SHapley Additive exPlanations) technique [131]. In their experiments conducted with the crisprSQL data set, Stortz *et al*. demonstrated the importance of both the sequence context and the chromatin accessibility for effective cleavage prediction. Niu et. al. [132] proposed the R-CRISPR deep learning model that encodes sgRNA target sequences into a binary matrix and then uses a CNN model as a feature extractor. Precisely, the authors applied a Rep-VGG inference time body composed of a stack of 3$\times $3 convolutions and ReLUs [133] in the convolutional layers to extract relevant features. The CNN output was then passed to a bi-directional recurrent layers using an LSTM to get accurate off-target predictions. Fu *et al*. [134] introduced the MOFF off-target predictor based on the MOFF-target score function that is the sum of the multiplication of individual mismatch effect (IME), the combinatorial effect (CE), and the guide-intrinsic mismatch tolerance effect (GMTE), where GMTE is estimated from a dinucleotide CNN regression model. Two different encoding strategies: (1) mononucleotide encoding and (2) dinucleotide encoding were used to vectorize the input sequences. The tests conducted on a high-throughput allele-editing screen of 18 cancer hotspot mutations confirmed that MOFF significantly improves the selectivity and expands the application domain of Cas9-based allele-specific editing.

Concerning the on-target prediction, Shrawgi *et al*. [84] introduced DeepSgRNA, a deep learning architecture relying on CNN, to identify and predict RNA guides. The aim of their approach was to eliminate the need in manual feature construction, which improved the scalability of the approach. DeepSgRNA relies on hierarchical feature generation abilities of CNNs. In their experiments with the GenomeCRISPR data, the authors proved that DeepSgRNA was able to achieve state-of-the-art sgRNA prediction efficiency. Furthermore, we need to mention the pioneering work of Wang *et al*. [56], who compared several deep learning and conventional machine learning models to provide gRNA activity predictions for SpCas9 (eSpCas9(1.1)), Cas9-High Fidelity (SpCas9-HF1), and wild-type SpCas9 (WT-SpCas9) data. Feature engineering was performed using the effective Tree SHAP technique [131] that combines the SHAP values [135] with the XGBoost algorithm. The authors built two RNN and one CNN deep learning models, and trained them along with a linear regression, a L2-regularized linear regression, an XGBoost, and a multilayer perceptron. In their experiments, Wang *et al*. demonstrated that the RNN that received as input the sequence data with added biological features was able to outperform the other competing models. Their second best-performing model was the RNN that received as input sequence data only. Furthermore, Wang *et al*. developed a DeepHF website to ease the access to WT-SpCas9, eSpCas9(1.1), and SpCas9-HF1 indel data. The authors also applied SHAP with XGBoost and RNNs to assess the feature importance of the sequence input. Moreover, they used the Deep SHAP algorithm [135] to estimate the position-dependent gRNA nucleotide contribution to deep learning predictions. Based on the results of Wang *et al*., we can conclude that additional biological information and plausible reengineered features increase the predictive performance of deep learning models, leaving opportunities for future model enhancement. Elkayam and Orenstein [136] proposed the DeepCRISTL on-target prediction model, which can be considered as an improvement of DeepHF [56]. It is based on the use of the BLSTM (Bidirectional Long Short-Term Memory) and transfer learning techniques. To improve the prediction performance of DeepHF, the authors also considered some plausible biological features of the DeepHF data set. Elkayam and Orenstein used random hyperparameter search to carry out hyperparameter optimization of their DeepCRISTL-pre-train model, which allowed them to outperform the DeepHF model proposed by Wang *et al*. [56].

Finally, the CRISPRon and CRISPRoff deep learning models, based on CNN and gradient boosting regression trees, and the related interactive webserver were developed by Xiang *et al*. [123]. For their prediction, the authors used different position-specific sequence, position-independent, and thermodynamic features. They established that the gRNA–DNA binding energy is a major contributor in predicting the on-target activity of gRNAs. Using the CRISPRon model, one can compute the on-target efficiency of all possible gRNAs with NGG PAM sequences, for genes, genomic regions, or custom sequences. The CRISPRoff model allows users to compute the specificity of gRNAs with NGG PAM sequences. Moreover, by means of CRISPRoff, one can compute the relative likelihood of cleavage by CRISPR-Cas9 at off-target sites compared with the likelihood of cleavage at on-target sites.

Feature engineering has proven to be essential in many research fields involving machine learning predictions. We think that this aspect has not yet been fully explored in CRISPR/Cas9 and that future investigations should contribute to higher classification performance of both traditional machine learning and deep learning models applied to predict on- and off-target activities.

### Models relying on class rebalancing techniques

Training deep learning models with real-world CRISPR-Cas9 data is challenging because of a large natural imbalance existing between positive and negative samples. This leads to an imbalanced data classification problem with a much larger majority class and a much smaller minority class. Thus, the predictive models observe and learn more from samples of the majority class and, as a consequence, can fail to identify accurately samples from the minority class. This can negatively impact their overall predictive performance. In CRISPR/Cas9, the problem of data imbalance mainly applies to the task of off-target prediction.

In this context, we need to mention the work of Zhang *et al*. [77] and their DL-CRISPR deep learning model of off-target activity prediction, with data augmentation as a solution for the class imbalance problem. The authors first gathered data from two source types (i.e. from *in vitro* and cell-based experiments) to increase the size of the positive class samples (i.e. off-targets), and thus improve the model’s competency. Precisely, Zhang *et al*. proposed to increase synthetically the number of positive samples by rotating the sgRNA–DNA encoded images by 90, 180, and 270 degrees, respectively. Hence, the number of positive samples in the data set was quadrupled. The data were then passed to a four-layer CNN to perform the off-target prediction. The main finding of this work was that data augmentation was a critical step for improving the predictive performance of DL-CRISPR. Class rebalancing and data augmentation are still fast evolving domains. Alleviating data imbalance should boost the predictive performance of off-target prediction models as class imbalance remains one of the intrinsic properties of CRISPR-Cas9 data.

### Models relying on attention mechanism

Recent progress in the development of deep learning models using attention mechanism [137] has triggered interest in many research fields, including CRISPR-Cas9, where it has already provided some promising off-target specificity and on-target efficiency prediction results.

Recently, Zhang *et al*. [78] have implemented the CRISPR-IP off-target prediction model based on a CNN, a BLSTM, and an attention layer learning the sgRNA–DNA sequence pair features. CRISPR-IP combines the four following types of network layers: (i) the convolutional layer to learn local features, (ii) the recurrent layer to learn the context features of the sequences, (iii) the attention layer to learn global features from the attention score, and (iv) the dense layer to map the features to the sample label space. The authors also used a new type of encoding scheme to overcome the problem of information loss in the sequence encoding. Xiao *et al*. [138] designed AttCRISPR, a deep learning framework based on the attention mechanism for predicting on-target activity. The proposed approach relies on two attention modules, one spatial and one temporal, facilitating the model’s interpretability. AttCRISPR uses an ensemble learning strategy stacking encoding-based and embedding-based methods to improve its predictive performance. Liu *et al*. [79] analyzed two transformer-based neural networks, AttnToMismatch_CNN and AttnToCrispr_CNN, using cell-specific information of genes. Both models are similar, except that AttnToCrispr_CNN employs a linear regression at the final layer. AttnToMismatch_CNN and AttnToCrispr_CNN demonstrated competitive performance for both off-target sgRNA specificity prediction and on-target efficiency prediction. Furthermore, Liu *et al*. introduced a third model, called seqCrispr, which harbors an LSTM component and a CNN component in parallel to provide accurate on-target efficiency predictions. Finally, Zhang *et al*. [82] proposed two novel interpretable attention-based CNN models, called CRISPR-ONT and CRISPR-OFFT, designed for predicting sgRNA's on- and off-target activities, respectively. Their methodology emphasizes the importance of the feature explainability for obtaining accurate on- and off-target predictions. Interpretable attention-based CNNs were used to highlight how RNA-guide Cas9 nucleases could be used to investigate mammalian genomes.

We are confident that future promising methods involving attention mechanism and deep learning should soon emerge in the field.

Table 4 summarizes the most important recent deep learning models used for on- and off-target target prediction in CRISPR/Cas9.

**Table 4:** A summary of studies applying deep learning models for on and off-target prediction in CRISPR/Cas9.

Study	Year	Target	ML model(s)	Encoding	Data	Link for data/	Prediction metric/
		prediction	used			software	results
Chuai *et al*.	2018	Off-target	DeepCRISPR	One-hot	$0.68$ billion sgRNA	https://github.	Spearman: 0.246
[71]		and	(DCDNN)		sequences derived	com/bm2-lab/	AUROC: 0.804
		on-target			from 13 human cell	DeepCRISPR	AUPRC: 0.303
					lines were considered		
					to generate		
					DeepCRISPR data		
Lin *et al*.	2018	Off-target	CNN and FNN	One-hot	GUIDE-seq [43],	https://github.	AUROC: 97.2$\%$
[73]					CRISPOR [60]	com/Michael	for CNN
						Linn/off_target	AUROC: 97$\%$
						_prediction	for FNN
Xue *et al*.	2018	On-target	DeepCas9	One-hot	Wang [52],	https://github.	Spearman:
[129]			(1D CNN)		Doench V1 [54],	com/lje00006/	0.23-0.61
					Doench V2 [59],	DeepCas9	
					HCT116 [62],		
					Z_fish MM [63],		
					Z_fish VZ [64],		
					Z_fish GZ [65],		
					Chari [67],		
					Ciona[68],		
					Farboud [69]		
Liu *et al*.	2018	On-target	SeqCrispr	Embedding,	DeepCRISPR [71]	https://github.	Spearman: 0.77
[139]			(RNN+CNN	sgRNA–DNA	CRISPR-Cpf1 [140]	com/qiaoliuhub/	
			+transfer	binding		seqCrispr	
			learning)	melting			
				temperature +			
				DNase, CTCF,			
				RRBS, and			
				H3K4me3 peaks +			
				global gene			
				network properties			
Wang *et al*.	2019	On-target	DeepHF	Embedding +	DeepHF - the largest	github.com	Spearman: 0.867,
[56]			(RNN)	GC content +	gRNA on-target activity	/izhangcd	0.862, and 0.860
				position-specific	set for mammalian cells	/DeepHF,	
				features +		https://bio.	
				position-		tools/DeepHF	
				independent			
				features +			
				thermodynamic			
				features			
Shrawgi *et al*.	2019	On-target	DeepSgRNA	One-hot	GenomeCRISPR [55]	http://genomecrispr.org	Spearman: 0.82
[84]			(CNN, with				AUROC: 0.85
			Hierarchical				
			feature				
			generation				
			abilities)				
Liu *et al*.	2019	Off-target	AttnToMis	Embedding	DeepCRISPR [71]	https://github.	AUROC: 0.961,
[79]			match_CNN			com/qiaoliuhub/	AUPRC: 0.071
			(Transformer			AttnToCrispr	
			+2dCNN)				
		Off-target	AttnToCrispr	Embedding			Spearman: 0.778
			_CNN				Pearson: 0.781
			(Transformer				MSE: 412 $\pm $ 27
			+CNN)				MSE: 412 $\pm $ 27
							
		On-target	seqCrispr	One-hot			Spearman: 0.765
			(LSTM+CNN).				Pearson: 0.760
							MSE: 442 $\pm $ 33
Dimauro *et al*.	2019	On-target	CRISPRLearner	One-hot	Farboud [69]	https://github.	Spearman:0.23
[141]			(deep CNN and		Wang [52],	com/pierclgr/	-0.69
			data		Doench V1 [54],	crisprlearner	
			augmentation)		Doench V2 [59],		
					HCT116 [62],		
					Z_fish MM [63],		
					Z_fish VZ [64],		
					Z_fish GZ [65],		
					Chari [67],		
					Ciona[68]		
Wang *et al*.	2019	On-target	CNN with	One-hot	Cas9,	https://github.	Spearman:
[142]			5layers+		eSpCas9,	com/biomed	0.582, 0.7105,
			transfer		Cas9 ($\Delta $recA)	Bit/DeepSgrna	0.360
			learning		[143]	Bacteria	
Aktas *et al*.	2019	Off-target	CNN, MLP,	One-hot	DeepCRISPR [71]	https://github.	Accuracy: 96.7$\%$
[144]		and	BLSTM			com/bm2-lab/	
		on-target				DeepCRISPR	
Kim *et al*.	2019	On-target	DeepSpCas9	One-hot	DeepSpCas9	http://deepcrispr.	Spearman: 0.73
[57]			(3 1D-CNN)			info/DeepSpCas9	
Liu *et al*.	2020	Off-target	CnnCrispr	Embedding	DeepCRISPR [71]	https://github.	AUROC: 0.957
[80]			(BLSTM	(GloVe embedding		com/LQYoLH/	AUPRC: 0.429
			and CNN)	model [81])		CnnCrispr	
Zhang *et al*.	2020	Off-target	DL-CRISPR	One-hot	A series of *in vitro*	https://github.	Accuracy:
[77]			(Data		and cell-based	com/yuu	98.57$\%$,
			augmentation)		assays were collected	uuzhang/	Sensitivity:
					to generate data	DL-CRISPR	95.13$\%$
					using a new data	_offtarget	
					augmentation method	_prediction	
Zhang *et al*.	2020	On-target	CNN-SVR	One-hot	DeepCRISPR [71]	https://github.	AUROC: 0.94
[145]						com/Peppags/	Spearman: 0.7
						CNN-SVR	
Chen *et al*.	2020	Off-target	DNA-BERT	Embedding	DeepCRISPR [71]	https://github.	AUROC: 0.993,
[146]			and			com/bm2-lab/	AUPRC: 0.594
			LightGBM			DeepCRISPR	Spearman: 0.276
Zhang *et al*.	2020	On-target	C-RNNCrispr	One-hot	DeepCRISPR [71]	https://github.	AUROC: 0.976
[147]			(CNN+RNN)			com/Peppags/	Spearman: 0.877
						C-RNNCrispr	
							
Trivedi *et al*.	2020	Off-target	Crispr2vec	One-hot	GUIDE-seq [43],	http://www.	Spearman: 0.60
[148]			(logistic		CIRCLE-seq [44]	rgenome.net/	AUROC: 0.91 on
			regression,			cas-offinder	unseen sgRNAs,
			SVM and DNN)				0.88 on the smart
Lin *et al*.	2020	Off-target	CRISPR-Net	One-hot	GUIDE-seq [43],	https://codeocean.	AUROC: 0.995
[74]			(LRCN)		Doench V2 [59],	com/capsule/	AUPRC: 0.317
					CRISPOR [60],	9553651/tree/v1	
					CIRCLE-seq [44],		
					SITE-Seq [45]		
Zhang *et al*.	2021	Off-target	CRISPR-OFFT	Embedding	Off-target data sets:	https://github.	AUROC: 0.97
[82]			(1d-CNN,		Digenome-seq [25],	com/Peppags/	AUPRC: 0.79
			attention)		GUIDE-seq [43],	CRISPRont-	
					BLESS [49, 50],	CRISPRofft	
		On-target	CRISPR-ONT	Embedding	On-target data sets:		AUROC: 0.865
					DeepHF [56],		
					Sniper-Cas9 [149],		
					SpCas9-NG [150],		
					xCas9 [151]		
Xiao *et al*.	2021	On-target	AttCRISPR	One-hot and	DeepHF [56]	https://github.	Spearman: 0.872
[138]			(Embedding	embedding		com/South-	
			-based Method)			Walker/AttCRISPR	
Charlier *et al*.	2021	Off-target	FNN, CNN,	One-hot	GUIDE-seq [43],	https://github.	AUROC: 0.995
[75]			RNN, RF,		CRISPOR [60]	com/dagrate/	AUC PR1: 0.949
			NB, LR.			dl-offtarget	Accuracy: 99.9$\%$
Stortz *et al*.	2021	Off-target	piCRISPR	One-hot +	crisprSQL [152]	https://github.	AUROC: 0.983
[130]			(RNN, CNN)	physically informed		com/florianst/	AUPRC: 0.978
				features: the target-		picrispr	Spearman: 0.1
				guide, the target-			
				mismatch, the target-			
				mismatch-type			
				and the target-			
				OR-guide encoding			
Xiang *et al*.	2021	On-target	CRISPRon and	One-hot +	A pool of 12,000	https://rth.	CRISPRon:
[123]		and	CRISPRoff:	GC content +	gRNA oligos,	dk/resources/	Spearman: 0.91
		off-target	Gradient boosting,	position-specific	targeting 3,834	crispr/crisproff	
			regression trees,	features	human protein-coding	and	
			CNN	position-independent	genes included in	https://rth.	
				features +	CRISPRon database,	dk/resources/	
				thermodynamic	Kim *et al*. data set [153]	crispr/crispron	
				features			
Niu *et al*.	2021	Off-target	R-CRISPR	One-hot	GUIDE-seq [43],	https://codeocean.	AUROC: 0.991
[132]			(bi-directional		Doench V2 [59],	com/capsule/	AUPRC: 0.319
			recurrent		CRISPOR [60],	9553651/tree/v1	
			network)		CIRCLE-seq [44],		
					SITE-Seq [45]		
Vinod kumar *et al*. [154]	2021	Off-target	GCN-CRISPR Graph Convolution Network	One-hot	CRISPOR [60]	DeepCrispr: https://doi.org/10.1186/s13059-018-1459-4, CnnCrispr: https://doi.org/10.1186/s12859-020-3395-z	AUROC: 0.987
Zhang *et al*.	2022	Off-target	CRISPR-IP	One-hot	CIRCLE-Seq [44],	https://github.	AUROC: 0.982
[78]			(CNN, BLSTM)		SITE-Seq [45]	com/BioinfoVirgo/	AUPRC: 0.751
						CRISPR-IP	Accuracy: 0.990
Elkayam and	2022	On-target	DeepCRISTL	Embedding +	DeepHF [56],	https://github.	Spearman: 0.878
Orenstein [136]			(BLSTM + transfer learning)	GC content + position-specific features + position- independent features + thermodynamic features	CRISPRon [123]	com/OrensteinLab/DeepCRISTL	
Fu *et al*.	2022	Off-target	MOFF	One-hot	GUIDE-seq data [43],	https://github.	Spearman: 0.5
[134]			(two CNN		CHANGE-seq [51],	com/MDhewei/	
			regression		TTISS [155]	MOFF	
			models)				

RNN: Recurrent Neural Network, CNN: Convolutional Neural Network, FNN: Feedforward Neural Network, DCDNN: Deep Convolutional Denoising Neural Network, LSTM: Long Short-Term Memory, BLSTM: Bidirectional Long Short-Term Memory, LRCN: Long-term Recurrent Convolutional Network, SVR: Support Vector Regression.

## CONCLUSIONS AND OUTLOOK

AI methods have emerged as state-of-the-art approach in the field of genome editing. We reviewed recent applications of traditional machine learning and deep learning algorithms for prediction of on- and off-target activity in CRISPR/Cas9. We believe that our review paper can serve as a guideline for CRISPR/Cas9 practitioners willing to apply AI methods in genome editing.

### Main Conclusions

The main conclusions of our study are as follows:

- First, we highlighted the importance of sequence encoding for sgRNA–DNA on- and off-target prediction. Initial models implied straightforward one-hot sequence encoding of the sgRNA–DNA sequence pairs [73]. Subsequent sequence encoding schemes were introduced [75] demonstrating higher predictive performance. The latest efforts have been focusing on the supplementary information embedding with different channels reflecting insertions, deletions, and mismatches [74].
- Second, some recent works have demonstrated that the ensemble ML methods have generally outperformed non-ensemble ML methods [46, 95]. For instance, AdaBoost [95] and random forest [46] led to superior predictive performance than a standard logistic regression or an SVM [100].
- Third, recent studies have highlighted the importance of feature selection and feature engineering for accurate activity prediction in CRISPR/Cas9. New methodologies have been introduced to incorporate sequence information, such as gene melting temperature, molecular weight, or microhomology features, into the model's input [56]. Some works emphasize the need of automated feature learning and automated feature engineering to boost the performance of deep learning models [78].
- Fourth, we observed that most of publicly available data sets have incomparable numbers of positive and negative samples, thus leading to a class imbalance problem that has a negative impact on the performance of both traditional ML and DL models, especially when predicting off-targets. Recent papers propose to use data augmentation to increase the number of samples of the minority class [77] or to apply some standard re-sampling techniques, such as under-sampling [80], to mitigate the impact of data imbalance [76].
- Fifth, for sufficiently large data sets, DNNs have demonstrated their superior predictive performance in comparison with both scoring methods and conventional ML algorithms such as SVM, random forest, and XGBoost [56, 73–75]. However, for smaller data sets, simpler ML tools were sometimes able to outperform some of their DL counterparts [37].
- Sixth, attention-based DL models have been extensively used in some recent works in the field [82, 137, 138]. The attention mechanisms have been shown to increase the efficacy of the deep learning process [156–159]. The latest DL models that rely on recurrent neural networks and attention-based mechanism have demonstrated very promising prediction performances [79, 138].

### Research Gaps and Future Research Directions

Research gaps and future research directions related to the application of AI methods in genome editing include:

- Powerful deep learning models have a huge number of parameters that need to be tuned in the training process. Thus, to be effective, these models require large amounts of input data. Transfer learning started to be used in the field to leverage the problem of deep learning training on data sets with insufficient amount of training samples. For instance, Lin *et al*. and Charlier *et al*. [73, 75] have successively employed transfer learning to predict the off-target sequences in small data sets. Further experiments should show how the most appropriate larger data sets used for training could be selected. Moreover, it would be interesting to see whether some pretrained deep learning models could be effectively used for transfer learning in CRISPR/Cas9.
- DNNs usually stack several layers of different types, each of which often containing dozens of neurons. Thus, designing efficient deep learning network architectures and finding optimal sets of hyperparameters remain extremely important and challenging tasks [160, 161]. Various hyperparameter tuning techniques, which should be extensively tested with CRISPR/Cas9 data, include evolutionary strategies, random grid search, exhaustive grid search, and Bayesian optimization [76].
- Explainability and interpretability of DNNs have been a topic of interest for the past few years [76, 162]. Recent methodologies have been introduced to further address the lack of human-level explainability and interpretability in the field [163–165]. Future research in genome editing could fill the gap in understanding the nature of on- and off-target activities, which would be an important milestone in clinical applications.
- As we pointed out, some recent works in the field have focused on the use of features engineering to boost the predictive performance of machine learning models [56, 84]. Informative features such as epigenetic features, microhomology properties, or RNA fold score can be further exploited to increase the models accuracy. Convolutional layers of CNN and LRCN deep learning networks are able to discover useful features from sequences directly and independently, avoiding eventual biases introduced by hand-crafted features [74, 84, 132]. The use of the SHAP [135] (SHapley Additive exPlanations—this algorithm gives an explanation to the model’s behavior, connecting optimal credit allocation with local explanations using the classic Shapley values from game theory), Tree SHAP [131] (this algorithm calculates SHAP values for tree-based models), and Deep SHAP [135] algorithms (this is a high-speed approximation algorithm for SHAP values) is highly recommended to assess how each feature impacts the selected model.
- Uncertainty quantification is a key technique to improve the trustworthiness of predictions made by a trained network. This technique has become popular for evaluating uncertainty in various research fields [166–170]. There are two types of uncertainty: the aleatoric uncertainty that is an inherent property of the data distribution and the epistemic uncertainty that refers to the model’s uncertainty. This technique could be effectively applied in genome editing to improve the trustworthiness of on- and off-target predictions. Kirillov *et al*. [171] have recently designed one of the first methods that incorporates uncertainty into the final prediction. This method provides interpretable evaluation of Cas9–gRNA and Cas12a–gRNA specificity using deep kernel learning, predicting the cleavage efficiency of a gRNA with a corresponding confidence interval.
- Active learning is a semi-supervised technique in which a learning algorithm is used to label unlabeled data. An active learning algorithm uses an initial subset of labeled data for training. The algorithm then predicts the most appropriate labels for unlabeled data. This technique is of particular interest in biology because obtaining labeled data is often costly and time-consuming [172–174]. Active learning can be employed in genome editing in situations when unlabeled data are abundant, while accurate automatic or manual labeling is impossible.

**Key Points**

- We reviewed current knowledge regarding the use of supervised machine learning methods for on- and off-target prediction in CRISPR/Cas9.
- We highlighted the importance of the data pre-processing step including encoding of the sgRNA–DNA sequence pairs without any information loss, embedding supplementary data with different channels reflecting insertions, deletions and mismatches, and considering some additional sequence information such as gene melting temperature, molecular weight, or microhomology features.
- Most of CRISPR/Cas9 data sets have incomparable numbers of positive and negative samples, thus leading to a class imbalance situation that should be mitigated using either data augmentation or data re-sampling techniques, especially in the case of off-target prediction.
- When training data sets were large enough, DNNs have demonstrated their superior predictive performance in comparison with scoring methods and traditional machine learning algorithms. However, for benchmark purpose, the results obtained using state-of-the-art deep learning methods should be compared with those provided by some effective conventional machine learning algorithms, such as SVM, random forest, and XGBoost.
- We emphasized the importance of feature selection for accurate on- and off-target prediction in CRISPR/Cas9. Thus, the automated feature learning and automated feature engineering techniques should be used to boost the performance of deep learning models.

## Author Biographies

**Zeinab Sherkatghanad** is a PhD student at the Department of Computer Science of Université du Québec à Montréal (Montreal, Canada). Her research interests are in the fields of data mining, deep learning and computer vision.

**Moloud Abdar** is an Associate Research Fellow with the Institute for Intelligent Systems Research and Innovation at Deakin University, Australia. His research interests include machine learning, deep learning, computer vision, uncertainty quantification, data mining and medical image analysis.

**Jeremy Charlier** is a Research Fellow at the Department of Computer Science of Université du Québec à Montréal (Montreal, Canada). His research interests include the application of deep learning methods in the fields of bioinformatics and finance.

**Vladimir Makarenkov** is a Full Professor and Director of a graduate Bioinformatics program at the Department of Computer Science of Université du Québec à Montréal (Montreal, Canada). His research interests are in the fields of bioinformatics, artificial intelligence, and data mining. They include design and development of new bioinformatics algorithms and software, such as practical tools for analysis of CRISPR-Cas9 and high-throughput screening (HTS) data.
